# Two-dimensional Penta-BP_5_ Sheets: High-stability, Strain-tunable Electronic Structure and Excellent Mechanical Properties

**DOI:** 10.1038/s41598-017-02011-9

**Published:** 2017-05-25

**Authors:** Shijie Liu, Bo Liu, Xuhan Shi, Jiayin Lv, Shifeng Niu, Mingguang Yao, Quanjun Li, Ran Liu, Tian Cui, Bingbing Liu

**Affiliations:** State Key Laboratory of Superhard Materials, Jilin University, No. 2699 Qianjin Street, Changchun, 130012 P.R. China

## Abstract

Two-dimensional (2D) crystals exhibit unique and exceptional properties and show promise for various applications. In this work, we systematically studied the structures of a 2D boronphosphide (BP) monolayer with different stoichiometric ratios (BP_x_, x = 1, 2, 3, 4, 5, 6 and 7) and observed that each compound had a stable 2D structure with metallic or semiconducting electronic properties. Surprisingly, for the BP_5_ compounds, we discovered a rare penta-graphene-like 2D structure with a tetragonal lattice. This monolayer was a semiconductor with a quasi-direct band gap of 2.68 eV. More importantly, investigation of the strain effect revealed that small uniaxial strain can trigger the band gap of the penta-BP_5_ monolayer to transition from a quasi-direct to direct band gap, whereas moderate biaxial strain can cause the penta-BP_5_ to transform from a semiconductor into a metal, indicating the great potential of this material for nanoelectronic device applications based on strain-engineering techniques. The wide and tuneable band gap of monolayer penta-BP_5_ makes it more advantageous for high-frequency-response optoelectronic materials than the currently popular 2D systems, such as transition metal dichalcogenides and black phosphorus. These unique structural and electronic properties of 2D BP sheets make them promising for many potential applications in future nanodevices.

## Introduction

Two-dimensional (2D) materials exhibit fascinating electronic properties and show great potential for various applications, such as electronics, optoelectronics and solar cells. Research on 2D materials has rapidly progressed in the last decade. Graphene, a 2D honeycomb structure of carbon, typically shows a linear band crossing at the Fermi level, resulting in excellent electron mobility, which makes it particularly attractive for applications in ultrafast high-frequency photodetectors and graphene-based broadband optical modulators^1, 2^. However, graphene is a semi-metal with a zero band gap, which limits its applications in nanoelectronics and optoelectronic materials such as field-effect transistors because the transistor cannot be turned completely off^3, 4^. Therefore, it is critical to find a new 2D material with natural semiconducting properties or to open the band gap to extend the possible applications.

One approach is to find a natural semiconducting 2D material in other compounds, such as the popular transition metal dichalcogenides (TMDCs)^5, 6^ and black phosphorus^7, 8^. TMDCs are 2D materials with the formula MX_2_, where M is a transition metal element and X is a chalcogen, such as MoS_2_, MoSe_2_, WS_2_ and WSe_2_^5^. The corresponding monolayer structures have been synthesised experimentally, and all of them exhibit excellent properties different from those of their bulk counterparts^6, 9–12^. For example, in some semiconducting TMDCs, the bulk material usually has an indirect band gap, whereas the corresponding monolayer has a direct band gap. Bulk MoS_2_ is typically a semiconductor with an indirect band gap of approximately 1.3 eV, whereas the monolayer possesses a direct band gap of 1.8 eV^9^. This direct band gap of single-layer MoS_2_ also leads to the photoluminescence effect, which enables the application of this material in optoelectronic devices^13, 14^. Nevertheless, TMDCs and black phosphorus also have disadvantages; for example, their band gaps are mostly less than 2.0 eV, which results in their failure to respond to photons with wavelengths less than 620 nm, such as blue and ultraviolet (UV) light-emitting diodes (LEDs) and photodetectors^15^.

Another approach to further open the band gap of a structure involves modifying its basic configuration because the properties are closely related to the structural configurations. Graphene generally has a hexacyclic configuration^1^. Surprisingly, Zhang *et al*. recently reported a very rare penta-graphene starting from the pure pentacyclic configuration^16^. This penta-graphene is not only dynamically and mechanically stable but also has a wide band gap (3.25 eV), unusual negative Poisson’s ratio, and ultrahigh ideal strength. Subsequently, more attention has been paid to this approach, which has been extended to other systems, such as CN_2_, B–N, Si–H, B_2_C and AlN_2_^17–21^. A corresponding stable penta-2D structure with a wide band gap above 2.0 eV in each compound was observed. Therefore, it is necessary to find a suitable penta-2D material to obtain a large-band-gap semiconductor.

As a typical semiconducting material, boronphosphide (BP) has also attracted considerable attention. Experimentally, bulk BP is the only known stable compound in the B–P system^22^. Bulk BP exhibits many outstanding semiconducting properties with an indirect wide band gap, resulting in its wide applications in solid-state neutron detectors^23^. BP films have been synthesised on silicon carbide using chemical vapour deposition^24^. Theoretically, all the previously predicted 2D BP structures have been constructed from hexagonal configurations, and all of them have a band gap in the range of 1–1.8 eV^25–27^. To date, no 2D material with a band gap larger than 2.0 eV has been reported in the B–P system. Therefore, determining whether a 2D single-layer structure composed of pure pentagons with a larger band gap exists in the B–P system is of interest. Moreover, current experimental and theoretical studies have focused on the 1:1 ratio of B to P, and the search for a 2D structure for other compounds remains lacking. Hence, whether stable 2D monolayer structures with excellent properties exist in other ratios of B and P remains to be determined.

To address these issues, we systematically studied the structures of 2D BP monolayers with different stoichiometries, including BP, BP_2_, BP_3_, BP_4_, BP_5_, BP_6_ and BP_7_, using particle swarm optimisation (PSO) combined with *ab initio* molecular dynamics (MD) calculations. The simulation results indicate that each compound has a stable 2D structure. Surprisingly, for BP_5_, we observed a rare penta-graphene-like structure with a quasi-direct band gap of 2.68 eV. Moreover, the 2D materials with ratios of 1:3, 1:6, and 1:7 exhibited semiconducting properties with indirect band gaps of 0.8–2 eV, whereas the 2D materials with 1:2 and 1:4 ratios exhibited metallic properties.

## Results and Discussion

### Structural properties

Six different compositions of 2D BP compounds were considered using CALYPSO, including 1:1, 1:2, 1:3, 1:4, 1:5, 1:6 and 1:7. The simulation results indicated that each compound contains a series of 2D structures. Here, we focus on the 1:5 compound.

**Figure 1:**
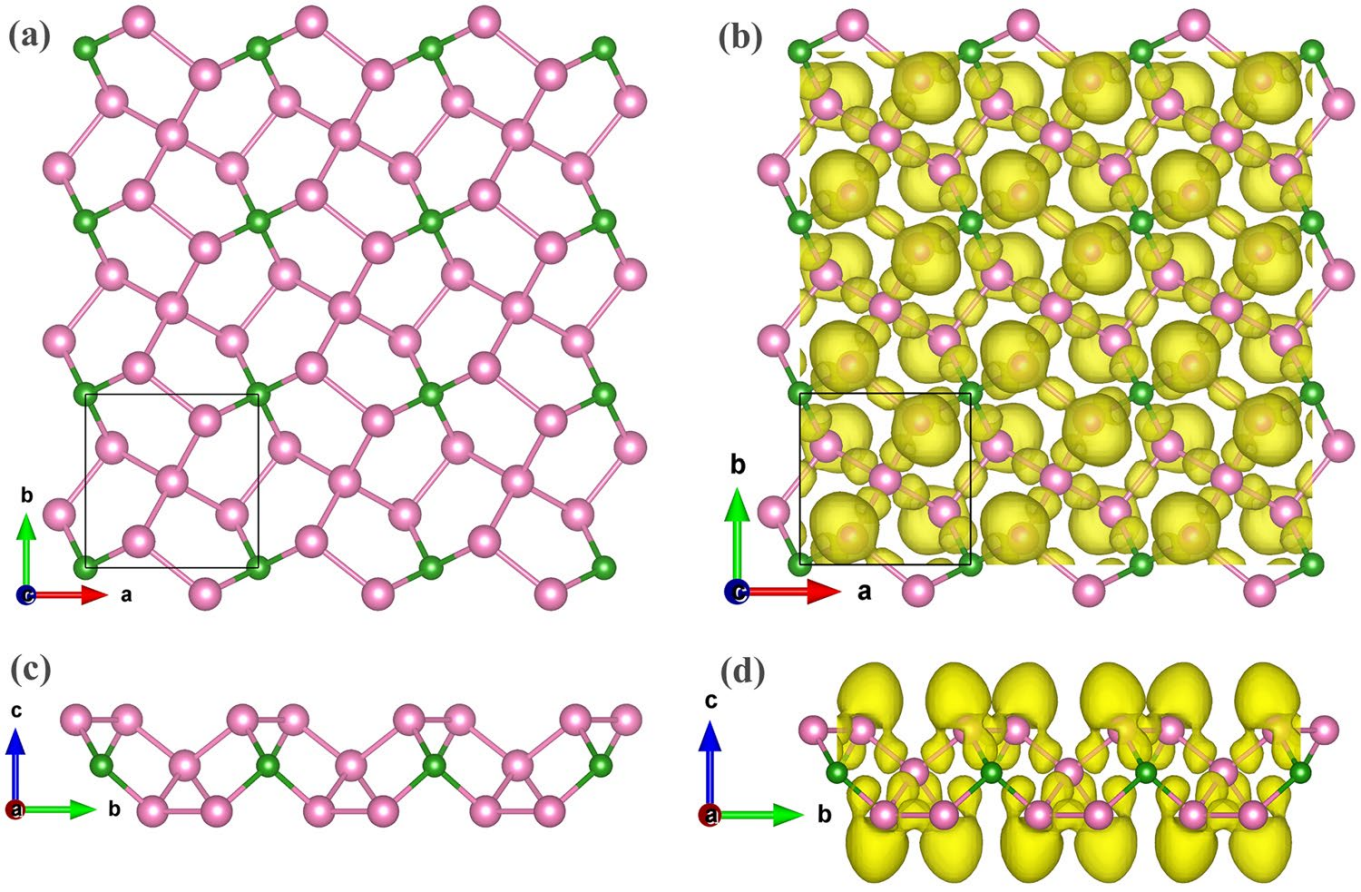
Top and side views of the (**a** and **c**) predicted structure and (**b** and **d**) corresponding ELF of penta-BP_5_ monolayer. The B and P atoms are denoted by pink and green balls, respectively. The isovalue of the ELF is 0.85.

Figure 1 shows the optimised 2D structures of the 1:5 compound. This penta-2D structure has a tetragonal lattice with a lattice parameter of a = b = 4.54361 Å and space group P-42_1_c (No. 114). This structure is similar to that of the previously reported penta-graphene structure, which possesses P-42_1_m symmetry (space group No. 113)^16^. As observed in the top view of penta-BP_5_ in Fig. 1a, the structure is constructed of pure pentagons, where each pentagon includes one B atom and four P atoms, forming the famous Cairo pentagonal tiling^16^. In the side view of penta-BP_5_ (Fig. 1c), buckling is observed. The “thickness” of this sheet is 2.50 Å, which is the vertical coordinate difference between P atoms in the top and bottom layers. The “thickness” of penta-BP_5_ is larger than that of penta-graphene (1.20 Å) and other penta-2D materials^17–21^. The lattice parameters and atomic positions are listed in Table S1.

In Fig. 1(b–d), the electron localisation function (ELF) was calculated using the Perdew–Burke–Ernzerhof (PBE) method. In the structure, all the B atoms are equivalent, forming a four-coordinated sp^3^ hybrid. There are two equivalent positions for the P atom, including the formation of four and three coordinates. Analysis of the ELF reveals that all the P atoms are also sp^3^ hybrid; however, there is alone pair of electrons for the three-coordinate case. It is an all-sp^3^ electronic structure that leads to the semiconducting properties^28^.

### Dynamical stability

**Figure 2:**
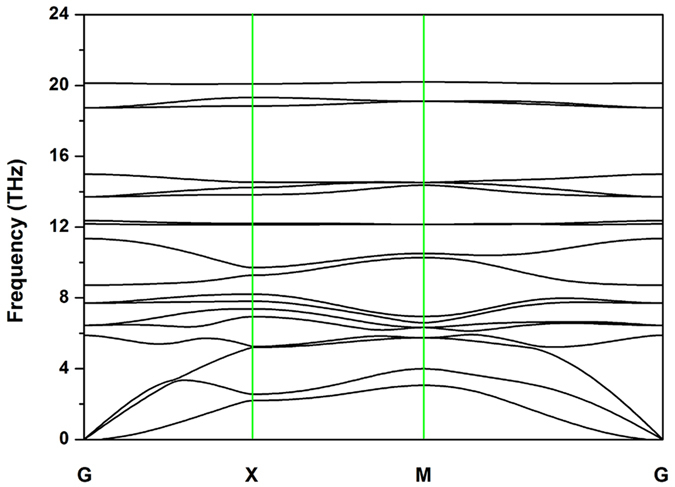
Calculated phonon dispersion of penta-BP_5_ monolayer.

To examine the dynamical stability of the 2D structure, we calculated the phonon spectra, as shown in Fig. 2. No imaginary modes are observed in the first Brillouin zone for penta-BP_5_, indicating its dynamical stability.

**Figure 3:**
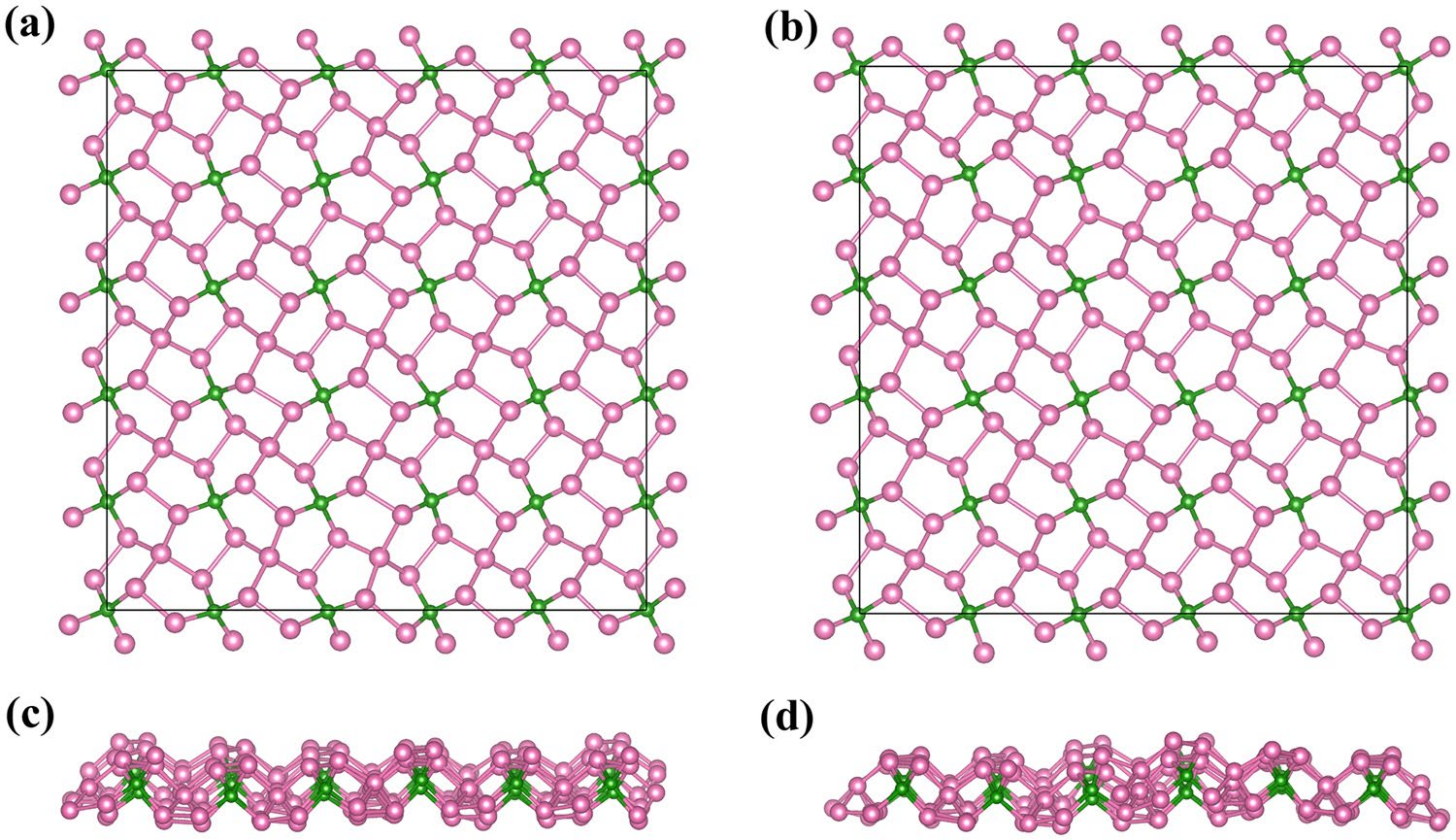
Top and side views of snapshots of the penta-BP_5_ monolayer equilibrium structures at 300 K (**a** and **c**) and 1000 K (**b** and **d**) after 10 ps in the MD simulations.

For the kinetically stable pentagonal BP_5_ monolayer, *ab initio* MD simulations were performed. A large supercell (5*5) was employed by heating the structure to 300 K, 450 K, and 1000 K. For each case, the simulation duration was 10 ps with a step of 1 fs. At the end of each simulation, the final structure was carefully examined. As observed in Fig. 3, the penta-BP_5_ could withstand temperatures as high as 1000 K. At high temperatures, the structure became slightly distorted. However, these distortions were not sufficient to destroy the B–P and P–P bonds, and the original structure could be restored by global optimisation. Therefore, the phonon calculations and MD simulations indicated that monolayer penta-BP_5_ possesses high dynamical stability.

### Mechanical properties

**Figure 4:**
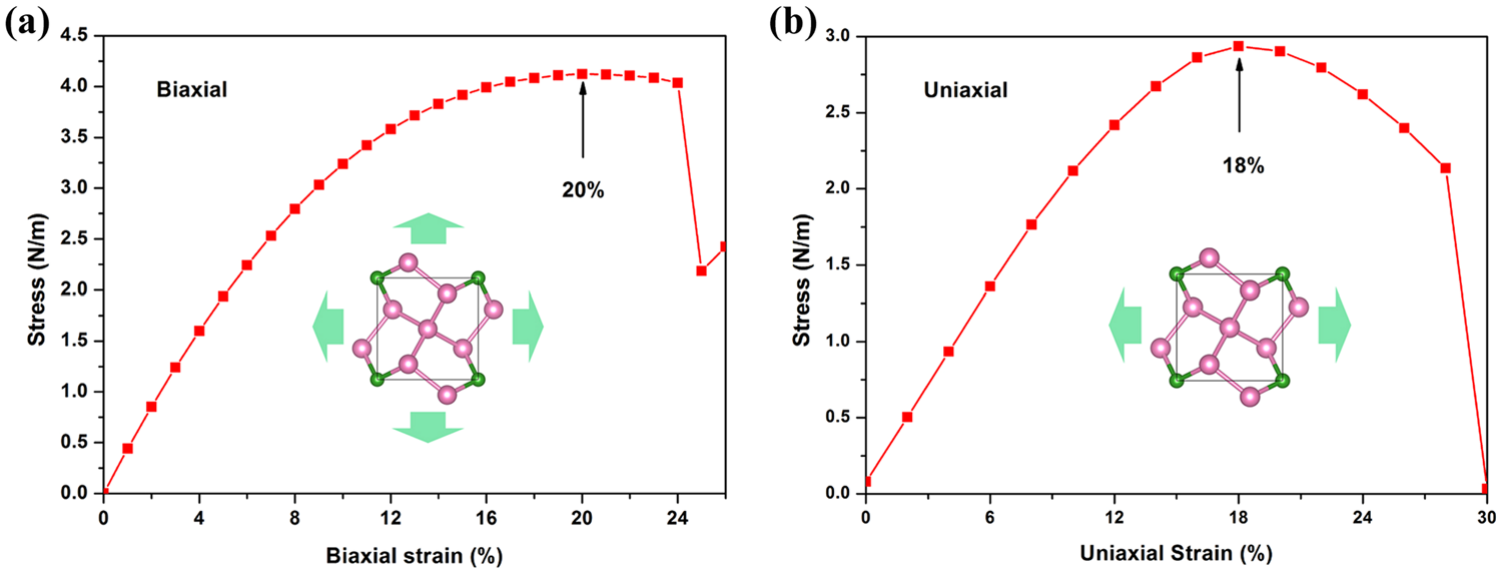
Stress in the penta-BP_5_ monolayer subjected to (**a**) biaxial and (**b**) uniaxial strain.

Excellent strain strength is indispensable for an ideal nanomaterial, and the stress–strain curve is an extremely important physical quantity that characterises the mechanical properties of a material. We added the effect of biaxial strain by changing the lattice parameters along the x- and y-axes. The strain is defined as (a − a_0_)/a_0_, where a and a_0_ are the lattice parameters of the phase with and without strain, respectively^19, 29^. The stress–strain curves of the penta-BP_5_ monolayer are presented in Fig. 4. The maximum external force that the system can withstand when the single layer is stretched is the maximum stress. Strain refers to the relative deformation of an object under external force, and its value characterises the plastic deformation capacity of the structure. As observed in Fig. 4a, the penta-BP_5_ monolayer can withstand a maximum stress of 4.1 N/m at a biaxial strain of 20%, indicating that the 2D material exhibits excellent mechanical properties comparable to well-known 2D materials such as graphene^30^ and MoS_2_^31^. Next, we investigated the case of uniaxial strain. As the x and y axes are equivalent, we introduced the uniaxial strain along the y-axis, and the x-directional lattice was allowed to relax freely^19^. Figure 4b shows the dependence of stress on uniaxial strain. We can see that maximum stress of the structure is 2.93 N/m at uniaxial strain of 18%, indicating that the structure has a high uniaxial tensile capacity.

### Electronic properties

**Figure 5:**
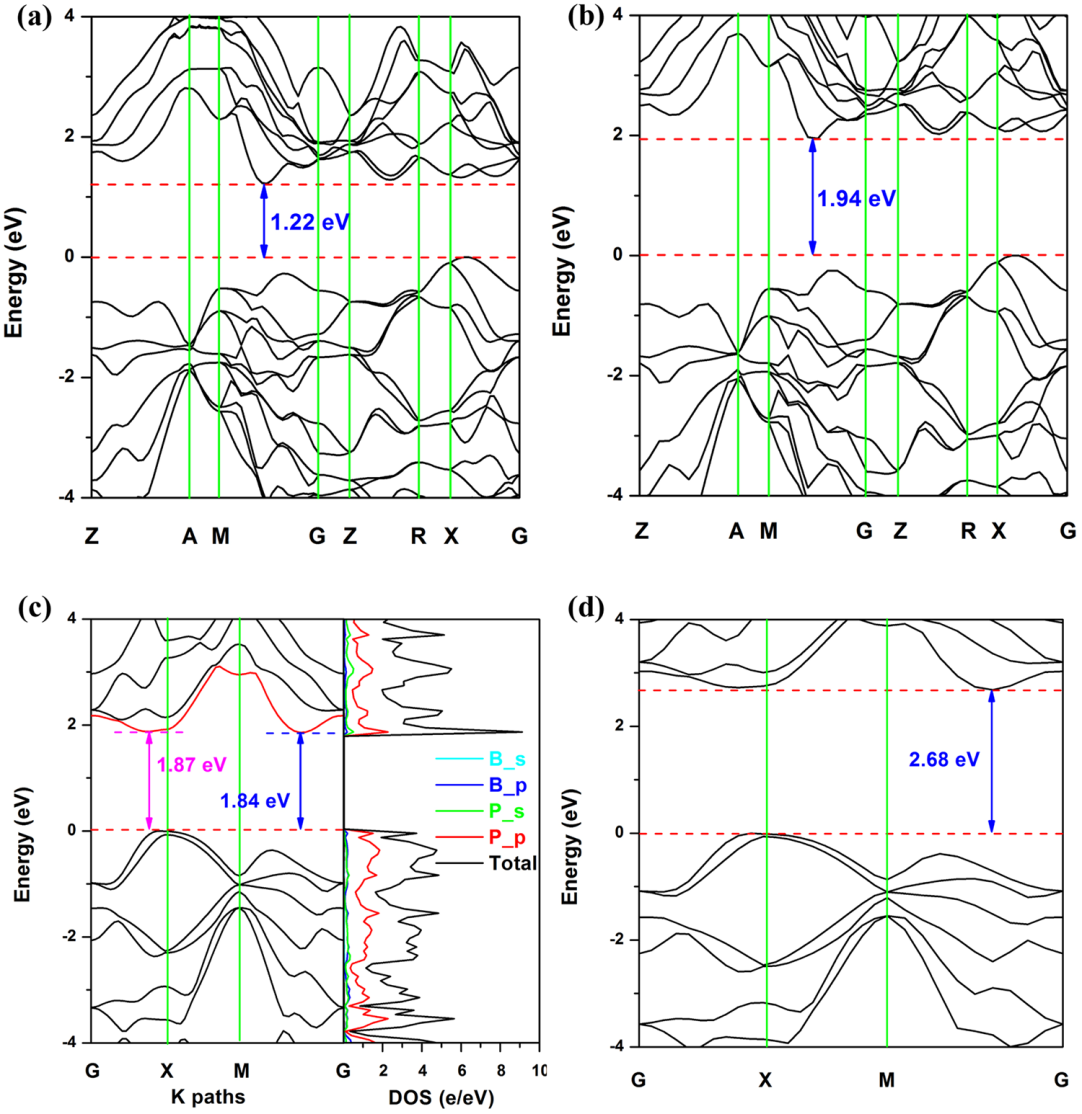
Band structures of penta-BP_5_ bulk using (**a**) PBE and (**b**) HSE06 methods. (**c**) Band structures (left) and orbital PDOS (right) of the penta-BP_5_ monolayer determined using PBE methods. (**d**) Band structures of penta-BP_5_ monolayer determined using HSE06 methods. The Fermi energy was set to zero.

We calculated the band structures and orbital projected density of states (PDOS) using the PBE method. The results indicate that the penta-BP_5_ bulk (Fig. 5a) is an indirect semiconductor with a band gap of 1.22 eV. Examination of the band structure of the monolayer (Fig. 5c left) reveals an interesting phenomenon. The valence band maximum (VBM) of the single-layer penta-BP_5_ lies at a point along the G–X line, and the conduction band minimum (CBM) lies at a point along the M–G line. The energy difference between the second-lowest point of the conduction band (along the G–X line) and the CBM is very small (0.03 eV), such that the monolayer structure can be considered a quasi-direct semiconductor with a band gap of 1.84 eV. A similar phenomenon is also observed in the Si–Ge super lattice system^32^. The PDOS of the monolayer is shown in Fig. 5c (right). Hybridization is observed in the 3s and 3p orbitals of the P atom and 2s and 2p orbitals of the B atom over the entire energy range, indicating that there is a covalent interaction between the P–P and B–P bonds.

Hybrid density functional (HSE06) calculations provide a better description of band gaps in semiconductors; as observed in Fig. 5b and d, the band gaps of the bulk and monolayer penta-BP_5_ are transformed to 1.94 and 2.68 eV, respectively. Therefore, 2D penta-BP_5_ is expected to be a promising candidate for photoelectric devices harvesting photons with wavelengths of less than 620 nm, such as blue and UVLEDs and photodetectors. Note that previous pentagonal layered structures have possessed either large or indirect band gaps, which limits their application in optical devices. Here, we report a novel penta-2D material with a suitable quasi-direct band gap for the first time.

**Figure 6:**
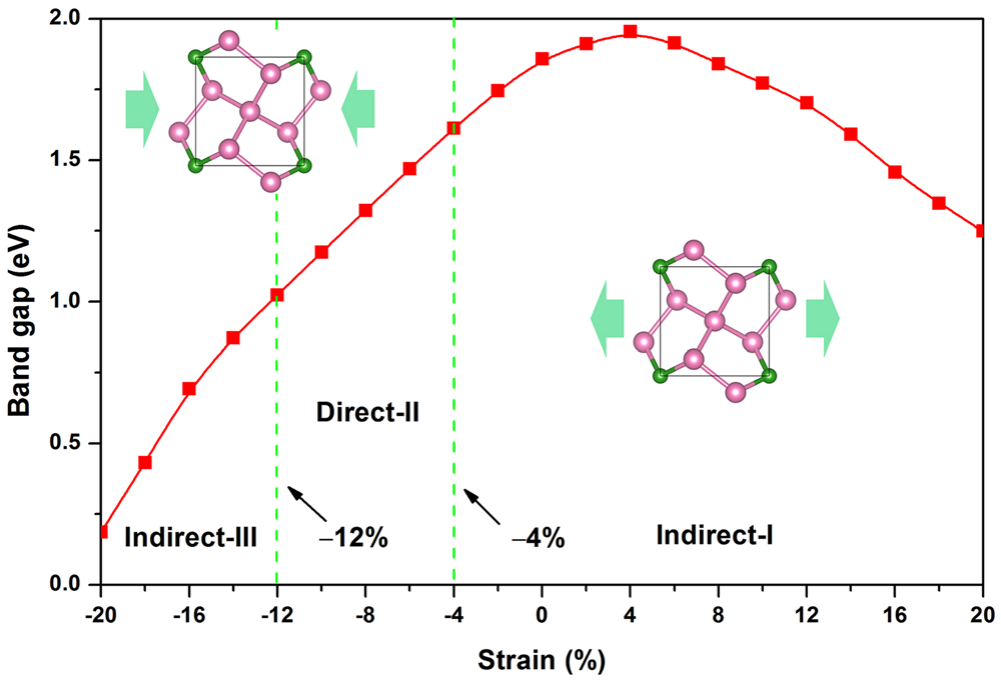
Variation of band gap with in-plane uniaxial strain for the penta-BP_5_ monolayer using the PBE method. The inset shows the direction of strains.

Strain technology is extensively applied to tune the band gap and electronic structures of semiconducting materials via lattice mismatch on the substrate, thermal expansion, or mechanical loading^33^. First, we considered the biaxial strain effect. We observed a transition from semiconductor to metal for penta-BP_5_ when the compressive strain was larger than −12%, and the metal properties were retained up to −20%. In the range of non-zero band gap, penta-BP_5_ is an indirect band gap semiconductor. The detailed results are presented in the supplementary materials (see Supplementary Fig. S1). In contrast to biaxial strain, uniaxial strain can further change the structure of a 2D material, resulting in new properties^34^. Therefore, we calculated the uniaxial strain to tune the electronic structure of the penta-BP_5_ monolayer. In Fig. 6, the band gap was calculated as a function of uniaxial strain (−20% to 20%) using the PBE exchange functional. The strain range can be divided into three parts: Indirect-I, Direct-II, and Indirect-III. In the strain range from −4% to 20% (Indirect-I), the monolayer is an indirect semiconductor. With continued expansion, the band gap exhibits an increasing tendency and reaches a maximum (1.95 eV) at 4% before decreasing nearly linearly to 20%. Interestingly, the monolayer undergoes a band-gap transition from quasi-direct to direct when a small compression (−4%) is applied. Moreover, this direct band gap can be maintained up to −12% (Direct-II). Due to the low phonon energy of electron excitation, this strain-tunable direct band gap semiconductor has obvious advantages in the application of optical devices. Up on further increasing the compression, the indirect band gap reappears and is retained until −20% (Indirect-III).

**Figure 7:**
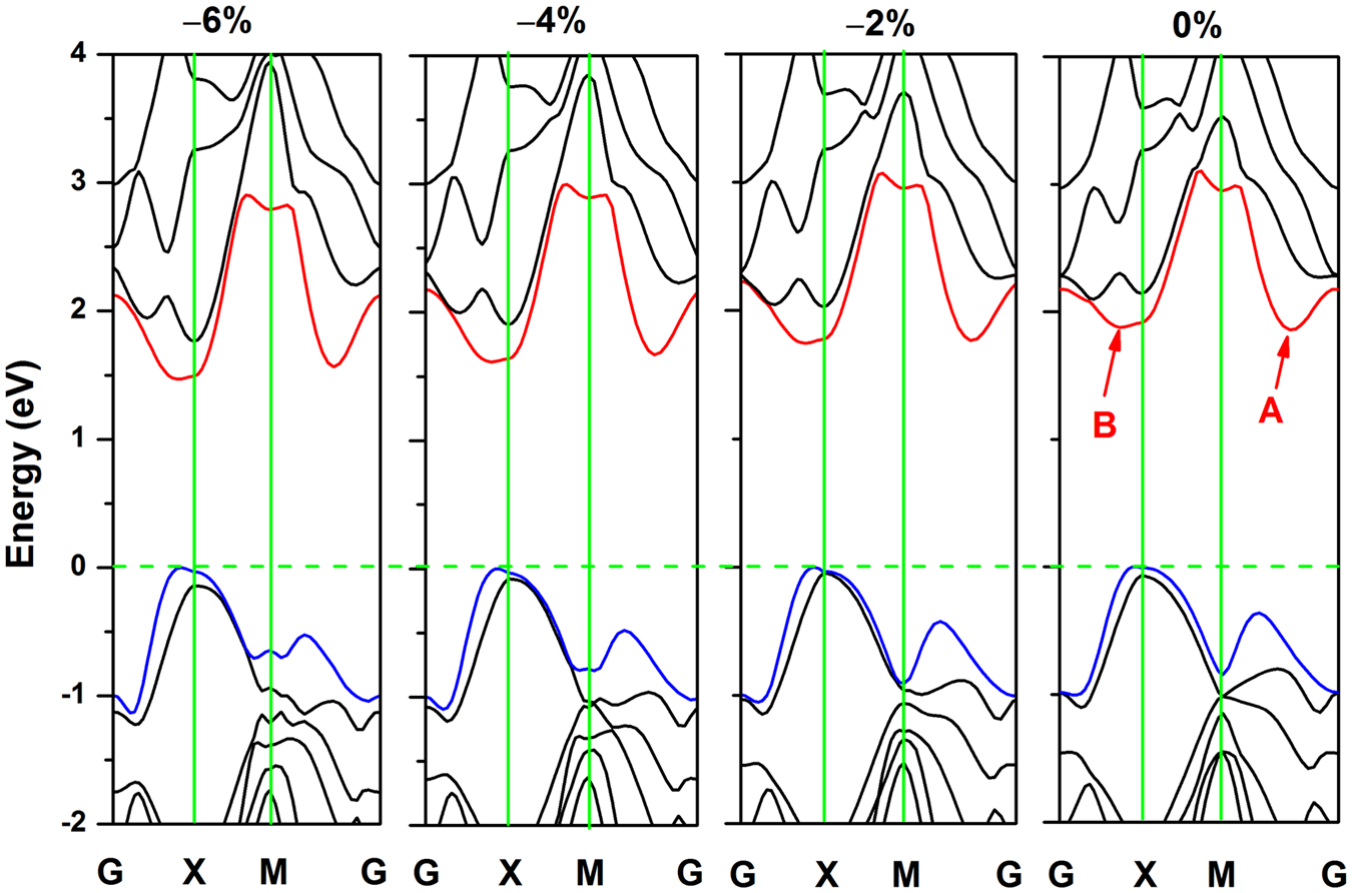
Strain-manipulated direct-to-indirect band gap in a 2D penta-BP_5_ monolayer with compressive strains of 0%, −2%, −4% and −6%. The Fermi energy was set to zero.

Next, we mainly discuss the physical mechanism of the band gap transition from quasi-direct to direct. The band structures with different compressive strains are shown in Fig. 7. For convenience, we labelled the CBM point and second-lowest point in energy at 0% strain as A and B, respectively. When not subject to external forces, the structure exhibited the properties of a quasi-direct band gap semiconductor, that is, the energy of point A is slightly smaller than that of point B. Closer inspection reveals that with increasing compressive strain, the energies of both point A and B decrease. However, the energy of point A decreases more rapidly and is considerably lower than that of point B at a compressive strain of −2%. In addition, the VBM and point A of the structure have similar K-space coordinates. These phenomena indicate that the structure begins to transform into a direct band gap semiconductor when compressive strains are applied. However, the energy of the VBM at state X slightly changes. Therefore, the band gap is mainly determined by shifts of the CBM. Overall, the entire conduction band has a tendency to approach the Fermi surface, which leads to the decrease of the band gap of the monolayer material under the compressive stress. At −4%, the penta-BP_5_ completely transforms into a direct band gap semiconductor with a band gap of 1.59 and 1.99 eV based on the PBE and HSE06 methods, respectively. This appropriate direct band gap makes this monolayer a promising candidate for solar cell materials.

In addition, we also studied other compounds of the BP system, including 2D structures with B to P ratios of 1:1, 1:2, 1:3, 1:4, 1:6, and 1:7. The results indicated that the 1:1 compound has a graphene-like structure, which is consistent with previous studies^25–27^. For the other compounds, we observed a series of stable 2D layered structures in each compound. The 2D materials for the 1:2 and 1:4 compounds exhibited metal properties, whereas those for the 1:3, 1:6, and 1:7 compounds exhibited semiconductor characteristics. All the 2D monolayer structures were dynamically stable, as confirmed by phonon dispersions. These 2D semiconducting materials were observed to be indirect band gap semiconductors with a band gap range of 0.8–2.0 eV (HSE06 calculations), indicating that these monolayer structures can be used as optical devices. The optimised structures and corresponding phonon spectral and band structures are presented in Supplementary Figs S2–S6.

## Conclusions

In summary, we performed a systematic search for stable 2D materials in the B–P system using the *ab initio* PSO methodology, including compounds with B to P ratios of 1:1, 1:2, 1:3, 1:4, 1:5, 1:6 and 1:7. A stable 2D structure was observed for each compound. Surprisingly, we observed a rare penta-graphene-like structure, penta-BP_5_, which is a semiconductor with a quasi-direct band gap of 2.68 eV (HSE06 method). The absence of an imaginary mode in the phonon spectrum and a high melting point indicated that the penta-BP_5_ monolayer exhibits good dynamical stability. Stress–strain calculations demonstrated that the penta-BP_5_ monolayer exhibits excellent mechanical stability with breaking biaxial and uniaxial strains above 20% and 18%, respectively. More importantly, analysis of the strain effect revealed that small uniaxial strain can trigger a quasi-direct to direct band gap transition in the penta-BP_5_ monolayer, whereas moderate biaxial strain can cause the penta-BP_5_ to transition from a semiconductor to a metal. This wide and tuneable band gap of monolayer penta-BP_5_ makes this structure more advantageous in high-frequency-response optoelectronic materials than the currently popular 2D systems, such as TMDCs and black phosphorus. In addition, the 2D materials in BP_3_, BP_6_, and BP_7_ have semiconducting properties with an indirect band gap of 0.8–2.0 eV, whereas the 2D materials in BP_2_ and BP_4_ have metallic properties. These unique structural and electronic properties of 2D B–P sheets make them promising for many potential applications in future nanodevices.

## Methods

The search for an energetically stable 2D B–P monolayer was performed by considering various stoichiometries of BP_x_ (x = 1, 2, 3, 4, 3, 4, 5, 6 and 7) using simulation cells containing up to four formula units. Structure searches for all stoichiometries were performed using the PSO methodology as implemented in the CALYPSO code^35, 36^. Total energy calculations, geometrical optimisations, and electronic properties were computed using the Vienna Ab Initio Simulation Package (VASP) program^37^. Exchange and correlation of the electrons were treated by the generalised gradient approximation (GGA) with the PBE^38^. The B and P potentials have 2s^2^2p^1^ and 3s^2^3p^3^ as valence states, respectively, and the cutoff energy of the plane waves was 500 eV. In all the 2D structures, a vacuum distance of 20 Å was used to separate the periodic images in the perpendicular direction. All the structures were fully relaxed, and the energy was converged to 1 meV per atom. To obtain highly accurate electronic structures, a hybrid HSE06 method was used^39^. The first-principles MD simulations lasted 10 ps with a time step of 1 fs with the canonical ensemble (NVT). Phonon spectra were calculated using the Phonopy code^40^.

## Electronic supplementary material

Supplementary information
