# Supplementary material for: Two-dimensional Penta-BP5 Sheets: High-stability, Strain-tunable Electronic Structure and Excellent Mechanical Properties
Source: Sci Rep. 2017 May 25;7:2404. doi: 10.1038/s41598-017-02011-9 (PMC5445080; doi:10.1038/s41598-017-02011-9)
Supplement: Supplementary file 1 — Supplementary information [file 41598_2017_2011_MOESM1_ESM.pdf]

Supplementary Information

Two-dimensional Penta-BP_5 Sheets: High-stability, Strain-tunable Electronic Structure and Excellent Mechanical Properties

Shijie Liu, Bo Liu, Xuhan Shi, Jiayin Lv, Shifeng Niu, Mingguang Yao, Quanjun Li, Ran Liu, Tian Cui, and Bingbing Liu*

State Key Laboratory of Superhard Materials, Jilin University, No. 2699 Qianjin Street, Changchun 130012, P.R. China,

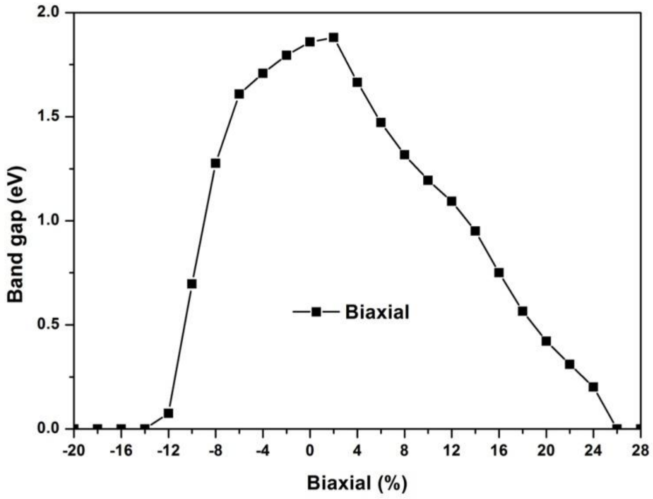

Fig. S1 Band gaps of the single-layer penta-BP_5 as a function of biaxial strain based on the PBE calculations.

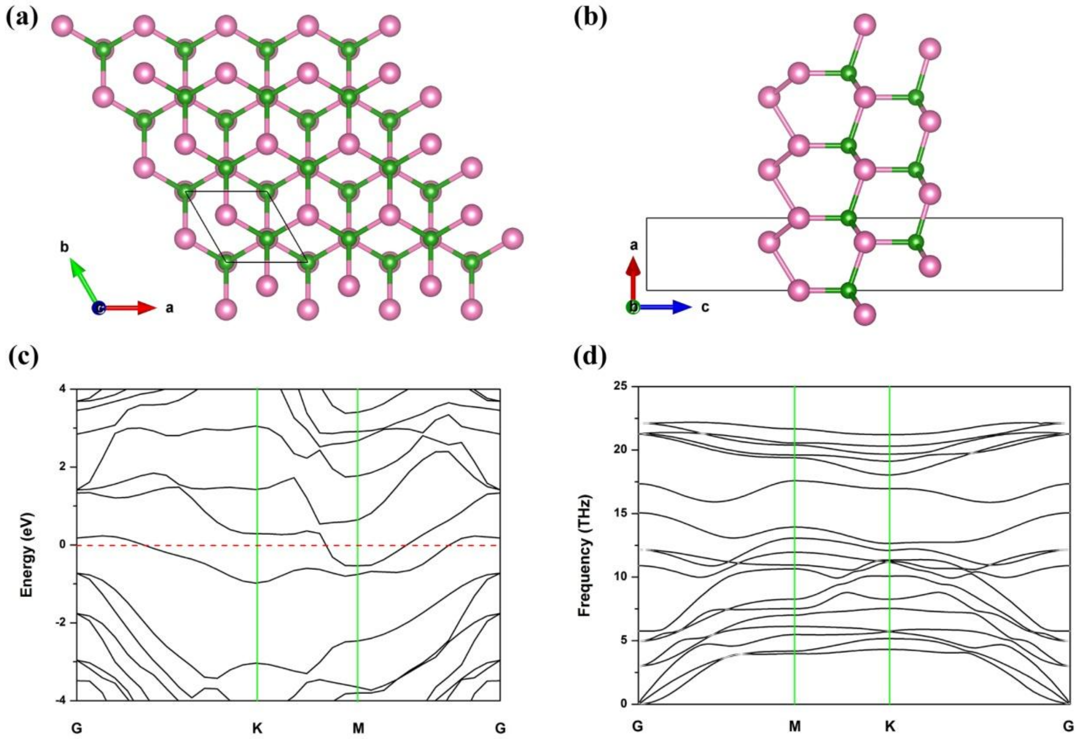

Fig. S2 (a) Top and (b) side view of the 2D monolayer of the 1:2 compound. (c) The calculated band structure by HSE06 method and (d) phonon dispersion of the corresponding monolayer. This structure can be seen as consisting of three-layer gaphene-like structure bonded with one and another. In the side view, from top to bottom, the first layer is composed of entirely P atoms; the second and third layers are composed of B and P atoms with the ratio of 1: 1.

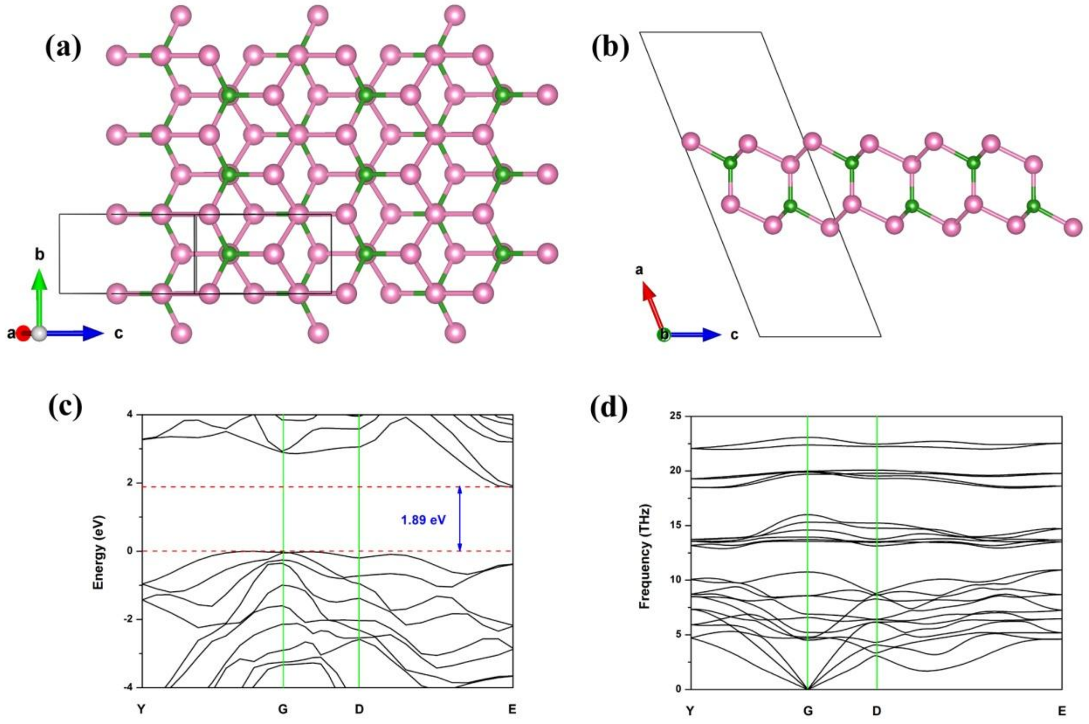

Fig. S3 (a) Top and (b) side view of the 2D monolayer of the 1:3 compound. (c) The calculated band structure by HSE06 method and (d) phonon dispersion of the corresponding monolayer. This structure can be seen as consisting of two-layer equivalent gaphene-like structures bonded with each other. In the side view, each layer is composed of B and P atoms with the ratio of 1: 3.

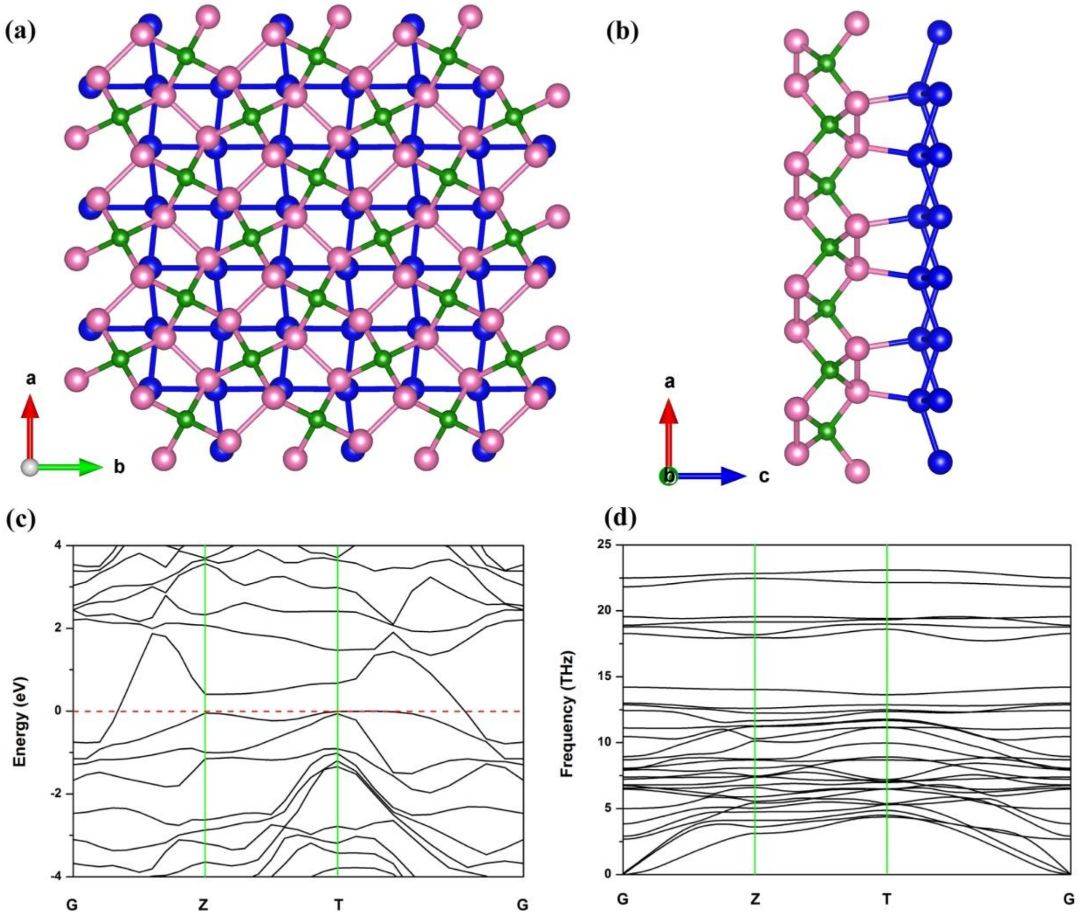

Fig. S4 (a) Top and (b) side view of the 2D monolayer of the 1:4 compound. (c) The calculated band structure by HSE06 method and (d) phonon dispersion of the corresponding monolayer. This is a very interesting structure. As shown in the side view, this structure consists of two parts, namely, a layer composed of pure pentacyclic configurations and a network of P atoms.

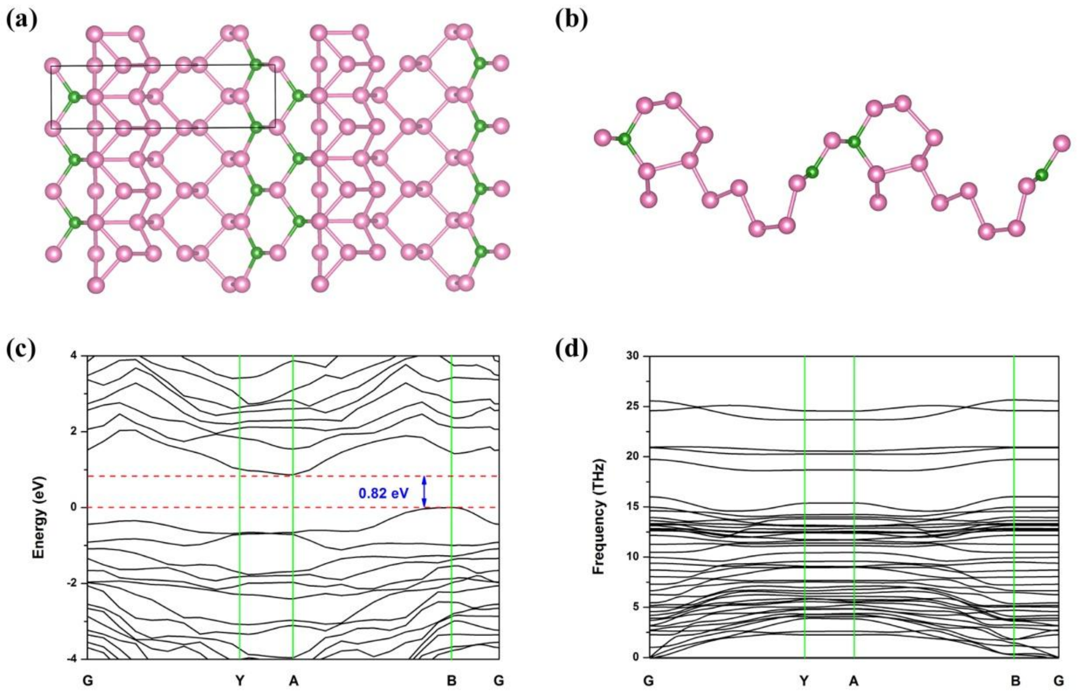

Fig. S5 (a) Top and (b) side view of the 2D monolayer of the 1:6 compound. (c) The calculated band structure by HSE06 method and (d) phonon dispersion of the corresponding monolayer. The structure has a peculiar configuration, in which a nanotube-like structure is included.

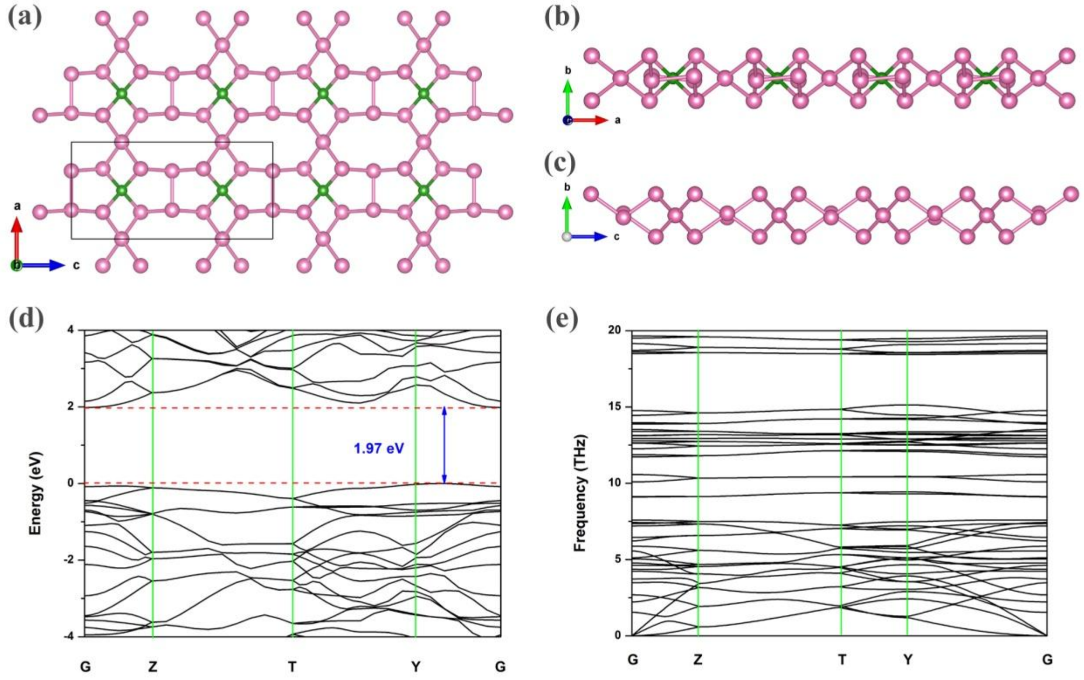

Fig. S6 (a) Top and (b and c) side views of the 2D monolayer of the 1:7 compound. (d) The calculated band structure by HSE06 method and (e) the phonon dispersion of the corresponding monolayer. This is a very novel structure. From the side view, we can see that this structure is composed of four rings, five rings and eight rings, respectively.

Table S1. Structural parameters of the bulk penta-BP_5.

Space group	Lattice parameters (Å)	Wyckoff	Atomic coordinates (fractional)			
$P\text{-}42_1c$	a= 4.5598	8e	P	0.19235	0.65230	0.37839
	c= 10.2693	2b		0.00000	0.00000	0.50000
		4a	B	0.00000	0.00000	0.00000
